# Nano-Strategies to Target Breast Cancer-Associated Fibroblasts: Rearranging the Tumor Microenvironment to Achieve Antitumor Efficacy

**DOI:** 10.3390/ijms20061263

**Published:** 2019-03-13

**Authors:** Marta Truffi, Serena Mazzucchelli, Arianna Bonizzi, Luca Sorrentino, Raffaele Allevi, Renzo Vanna, Carlo Morasso, Fabio Corsi

**Affiliations:** 1 Department of Biomedical and Clinical Sciences “L. Sacco”, Università degli studi di Milano, via G. B. Grassi 74, 20157 Milano, Italy; marta.truffi@unimi.it (M.T.); serena.mazzucchelli@unimi.it (S.M.); arianna.bonizzi@unimi.it (A.B.); luca.sorrentino1@unimi.it (L.S.); raffaele.allevi@unimi.it (R.A.); 2 Nanomedicine and Molecular Imaging Lab, Istituti Clinici Scientifici Maugeri IRCCS, via Maugeri 4, 27100 Pavia, Italy; renzo.vanna@icsmaugeri.it (R.V.); carlo.morasso@icsmaugeri.it (C.M.); 3 Breast Unit, Surgery Department, Istituti Clinici Scientifici Maugeri IRCCS, via Maugeri 4, 27100 Pavia, Italy

**Keywords:** cancer-associated fibroblasts, tumor microenvironment, nanoparticles, breast cancer, antitumor efficacy

## Abstract

Cancer-associated fibroblasts (CAF) are the most abundant cells of the tumor stroma and they critically influence cancer growth through control of the surrounding tumor microenvironment (TME). CAF-orchestrated reactive stroma, composed of pro-tumorigenic cytokines and growth factors, matrix components, neovessels, and deregulated immune cells, is associated with poor prognosis in multiple carcinomas, including breast cancer. Therefore, beyond cancer cells killing, researchers are currently focusing on TME as strategy to fight breast cancer. In recent years, nanomedicine has provided a number of smart delivery systems based on active targeting of breast CAF and immune-mediated overcome of chemoresistance. Many efforts have been made both to eradicate breast CAF and to reshape their identity and function. Nano-strategies for CAF targeting profoundly contribute to enhance chemosensitivity of breast tumors, enabling access of cytotoxic T-cells and reducing immunosuppressive signals. TME rearrangement also includes reorganization of the extracellular matrix to enhance permeability to chemotherapeutics, and nano-systems for smart coupling of chemo- and immune-therapy, by increasing immunogenicity and stimulating antitumor immunity. The present paper reviews the current state-of-the-art on nano-strategies to target breast CAF and TME. Finally, we consider and discuss future translational perspectives of proposed nano-strategies for clinical application in breast cancer.

## 1. Introduction

**Figure 1:**
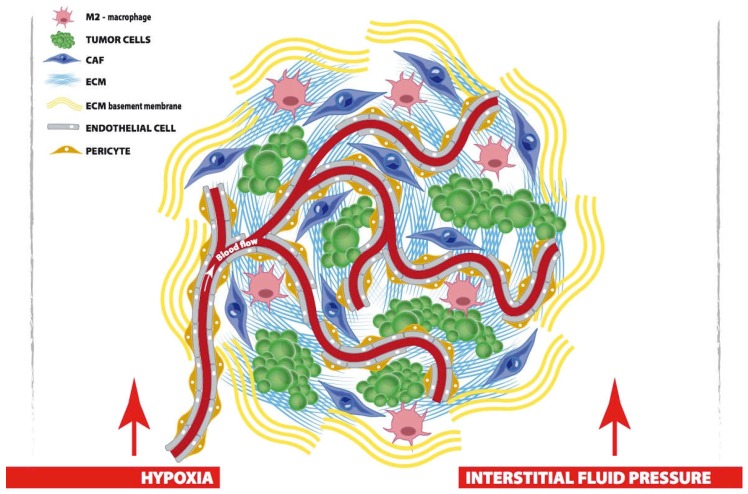
In desmoplastic cancer, tumor cells share their niche with CAF, macrophages, blood vessels and perivascular cells, all of which contribute to arrange the TME. First, CAF actively remodel cellular and matrix components of the TME; M2-polarized macrophages promote metastases and generate an immunosuppressive habit; endothelial cells and pericytes favor tumor angiogenesis and handle oxygen supply. Finally, in the niche, interstitial ECM supports tumor architecture and regulates drug penetration. Surrounding the tumor, ECM basement membrane acts as a barrier toward migration. Both ECM and blood vessels contribute to increase interstitial fluid pressure and tumor hypoxia, which are big obstacles to tumor treatment.

In recent years, the focus of cancer research has shifted from cancer to cancer-related stroma [1,2]. Indeed, tumor microenvironment (TME) plays a key role in several processes related to cancer progression, including the acquisition of an invasive phenotype, cell migration capability, chemoresistance, protection from antitumor immune response and neoangiogenesis [3]. The crosstalk between cancer and TME has such a pivotal relevance, that nowadays the epithelial-to-mesenchymal transition (EMT) is recognized as the central moment of cancer progression from in situ to invasive disease [4,5]. EMT refers to the milieu of biological processes by which cancer cells gradually lose their epithelial hallmarks and acquire mesenchymal properties related to invasion of surrounding tissues and remodeling of the extracellular matrix (ECM) [6]. The final result of EMT is the capability of cancer cells to metastasize in distant sites where, again, proliferation of cancer cells up to clinically detectable metastases depends on the organ microenvironment—the “seed and soil” hypothesis [7]. Thus, cancer stroma not only support, but are rather protagonists of cancer progression (Figure 1). Therefore, it is not surprising that currently an increasing proportion of research is focusing on development of anti-cancer strategies targeted toward TME [8]. Moreover, another more pragmatic reason is exciting the interest on anti-stromal therapies: TME is much more genetically stable than cancer [9,10]. A paradigmatic example is breast cancer, which is genetically and phenotypically heterogeneous [11]. Therefore, targeting breast cancer cells or pathways is rather difficult, considering that they change continuously between patients and in the same patient. A double-acting strategy targeting both breast cancer cells and TME might be a key for success in the treatment of breast cancer, but innovative drug delivery systems are needed. Nanomedicine could respond to this clinical unmet need, providing smart delivery systems based on active targeting and internalization both in cancer and in TME cells [12,13]. Due to their size and versatility, nanoparticles have attracted interest in the field of anticancer medicine, and they have shown ability to deliver consistent amounts of drugs and control their release at the tumor site [14,15,16,17,18]. Interesting feature of nanoparticles and nano-systems is the possibility to chemically or genetically modify their surface with a variety of targeting moieties or active ligands in order to trigger specific direction and recognition of the biological target [19,20,21]. The aim of the present paper is to review the current state-of-the-art and future translational perspectives on nano-strategies to target breast cancer microenvironment.

## 2. Biological Hallmarks to Target Breast CAF

**Figure 2:**
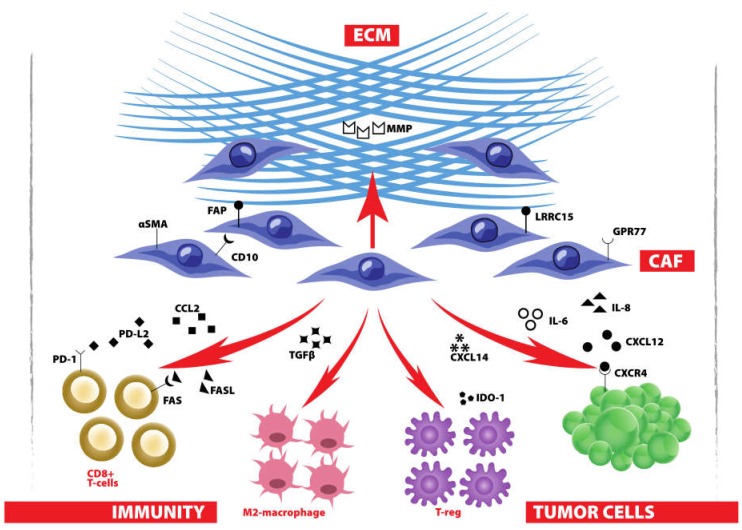
CAF and their essential role in the TME: CAF secrete cytokines to promote cancer growth and shape antitumor immunity; CAF control ECM composition to avoid drug penetration, favor tumor migration and mediate exclusion of antitumor immunity from the tumor.

Cancer-associated fibroblasts (CAF) are the most abundant cells of the tumor stroma and they critically influence cancer growth and progression through control of the surrounding TME (Figure 2). CAF produce and secrete a variety of growth factors and cytokines, including transforming growth factor β (TGFβ), vascular endothelial growth factor (VEGF), platelet-derived growth factor (PDGF), interleukins, as well as ECM components, particularly fibrillar collagens and fibronectins, and metalloproteinases (MMP) which support tumor growth, generate a physical barrier against drugs and immune infiltration and facilitate cancer invasion [1,22,23,24,25]. This contributes to generate the so-called reactive stroma and to induce a desmoplastic reaction in TME, which has been associated with poor prognosis in multiple carcinomas, including breast cancer [26,27,28]. Therefore, it is increasingly evident that effective anticancer therapies should tackle not only cancer cells but even such a tumor fortress composed by TME and orchestrated by CAF.

Previous studies have identified some phenotypical markers to detect and target pro-tumorigenic CAF in breast cancer. Among them, we can find markers of mesenchymal origin, such as αSMA or cell surface proteins associated with crucial biological functions of CAF, like fibroblast activation protein (FAP), which enzymatically remodel ECM and induce cancer cells migration [29], CD10, a zinc-dependent metalloproteinase [30], or G protein-coupled receptor 77 (GPR77), which activates VEGF expression and angiogenesis in hypoxic breast TME [31]. Leucine-rich repeat containing 15 (LRRC15) membrane protein was also found highly expressed on CAF in many solid tumors [32]. Exploitation of these markers to drive localization of novel therapeutics would be useful to eradicate CAF, and in some cases even to reprogram their biological functions. 

Additionally, CAF express large amounts of FAS ligand (FASL), which induces apoptosis of FAS-expressing CD8^+^ T cells, and programmed cell death 1 ligand 2 (PD-L2), which induces T cell anergy by interacting with the immune checkpoint molecule PD-1, thus avoiding anticancer immunity in the host [33]. CAF also secrete a lot of chemokine ligands (CXCL12/SDF1, CXCL14, CCL2, and others) that promote the proliferation of cancer cells and encourage the recruitment of tumor-associated macrophages, thereby contributing to immunosuppression [34,35,36,37]. In particular, the SDF1–CXCR4 interaction, together with the heat shock factor 1 (HSF1), generate an autocrine loop in CAF that drives the transcription of pro-tumorigenic cytokines and growth factors and supports rapid tumor growth [38,39]. Crucial signaling behind stromal communication with breast cancer cells is represented by the transcription factor STAT1, which is able to enlarge tumorigenicity and chemoresistance. Ablation of STAT1 in CAF may decrease cancer cell proliferation and reduce α-SMA+ reactive fibroblasts and ductal carcinoma in situ (DCIS)-like lesions in a mouse model of early breast cancer progression [38,39,40].

In recent years, some biological hallmarks of CAF have been exploited to design and study novel therapeutics and nano-therapeutics to remodel the TME and enhance the therapeutic activity of chemotherapy [8,41]. Some others have revealed insufficient specificity for CAF targeting or still require further preclinical research to achieve accessibility and exploitation for therapeutic purposes. Here, we resume and list some nano-systems that have been successfully explored in preclinical setting to target breast CAF and rearrange the breast TME (Table 1).

## 3. Active Nano-Systems for Breast CAF Disruption and Regulation

CAF are key actors in the restricted penetration of drug and nanodrug in the tumor tissue [56]. Indeed, CAF contribute to the biosynthesis and remodeling of the ECM and to the high tumor interstitial fluid pressure [57]. Therefore, the development of treatment’s strategy able to eradicate CAF could result in reduced collagen content in the ECM, leading to improved drug and nanodrug accumulation and diffusion. Many efforts have been made to the exploitation of CAF as a potential target for cancer therapy [8]. In particular, they have been involved the fibroblast activation protein (FAP), which is an integral membrane serine protease of the dipeptidyl peptidase subfamily selectively expressed by CAF. FAP is undetectable in the stroma of normal tissue [29], suggesting that the exploitation of FAP as a selective target of CAF could lead to specific and active delivery of cytotoxic drugs into these cells. Currently, three different nano-approaches have been investigated with the aim to specifically target CAF and improve therapeutic efficacy of anticancer treatments. They could be attributed to three different subcategories that will be described in detail in the following paragraphs. 

### 3.1. Nanoparticles for Photodynamic Therapy

In recent years, a novel antitumor approach called photodynamic therapy (PDT) has been exploited for cancer treatment with the aim to enhance nanoparticle’s tumor uptake and improve their therapeutic efficacy. This strategy consists in exposing the cells to non-toxic dose of light in presence of light-sensitive molecules, known as photosensitizer (PS). Conventional PDT uses non-targeting photosensitizer molecules and inflicts direct damage on tumor cells mainly generating reactive oxygen species (ROS) or acts indirectly by disrupting the vasculature via endothelial cells damage [58].

In this scenario, a very exciting idea is the possibility to perform a CAF-targeted PDT to modulate the microenvironment and promote the anticancer therapy avoiding systemic toxicities. Indeed, it is increasingly evident the crucial role of CAF in cancer progression due to their capability to isolate cancer cells from drugs and T-cytotoxic cell [59].

In this research area the most important studies have been performed by Zhen group, which exploited ferritin nanocages to deliver a photosensitizer to CAF in context of breast cancer [42,43]. They achieved CAF-targeted delivery of the photosensitizer thanks to the surface conjugation of an anti-FAP single chain variable fragment (scFv) antibody. Moreover, the combination of FAP-targeted delivery of the photosensitizer with a localized photoirradiation of the tumor, allowed the selective eradication of CAF. This treatment destroyed ECM and suppressed C−X−C motif chemokine ligand 12 (CXCL12) secretion by CAF, resulting in the significant improvement of CD8+ T-cell infiltration. This study suggests a novel approach to modulate TME through selective killing of CAF [42]. In another study, Zhen and colleagues combined the CAF-targeted PDT treatment with the administration of quantum dots (QDs), demonstrating that PDT treatment could be useful to improve QDs penetration. Indeed, this approach allowed CAF eradication in irradiated tumors and, in the meantime, promoted increase in nanoparticle’s tumor uptake, due to reduced amount of secreted collagen in the surrounding ECM [43]. Results from this study points out a potential role for CAF eradication in improving tumor drug penetration and documents a promising effective combination with chemotherapy. Finally, CAF-targeted PDT represents a great advance to modulate TME for optimal breast cancer management.

### 3.2. Nanoparticles for Cytotoxic Delivery

Another approach to disrupt CAF is represented by the exploitation of CAF destroying nanoparticles. They have been designed to deliver chemotherapeutics or drugs with specific cytotoxicity against CAF. Ji’s group proposed a double acting nanomaterial, which exploits the CAF targeting capability and an efficient cell penetration to improve chemotherapeutic drug delivery for the treatment of human prostate cancer model [60]. The researchers designed and synthesized a novel cell-penetrating peptide (CPP) based on an amphiphilic peptide (C2KKG2R9) linked by the hydrophobic tail to a cholesterol molecule monomer. Monomers of this CPP linked to a cholesterol molecule self-assembled into a core-shell structured peptide nanoparticles (PNP). PNP was then loaded with the antitumor drug doxorubicin (DOX) and the surface of the resulting DOX-loaded PNP (PNP-D) was modified with an anti-FAP monoclonal antibody to specifically recognize CAF. The anti-FAP antibody displayed CAF specificity, while the presence of CPP/cholesterol and DOX enhanced drug penetration and cytotoxicity, respectively [60].

In order to target CAF, another approach exploited a novel cleavable amphiphilic peptide (CAP), which is specifically responsive to FAPα, expressed on CAF surface [44]. In aqueous solution, CAP monomers readily self-assembled into nanofibers, which could be loaded with hydrophobic chemotherapeutic drugs, to obtain drug-loaded spherical nanoparticles (CAP-NP). Once they reached the tumor stroma, CAP-NP rapidly disassembled upon cleavage by FAPα and released the drug. This strategy was effective in the treatment of prostate, breast and pancreatic tumor models containing or recruiting FAPα+ CAF; it disrupted the stromal barrier and enhanced local drug accumulation. In case of breast cancer model, it has to be noted that MCF-7 breast tumor cell line was FAPα−negative but ultimately formed tumors that exhibited positive FAPα expression. Despite effective, the proposed approach may be possible with hydrophobic drugs only, as demonstrated in case of treatment of breast cancer-bearing mice with CAP-NP loaded with paclitaxel [44].

Recently, a peptide derivative nanofiber (C16-GNNQQNYKD-OH) has been designed and synthesized. It is able to self-assemble into long filaments entrapping losartan molecules inside a hydrogel exploitable for localized drug delivery [45]. Following a topical intratumor injection in mice model of aggressive triple negative breast cancer, this hydrogel induced inhibition of collagen I synthesis by CAF. Moreover, its combination with chemotherapeutic drug resulted in the amplification of the therapeutic efficacy [45].

Otherwise, in a work published by Chen and colleagues, nanoliposomes loaded with Navitoclax have been exploited for the target delivery of a small molecule inhibitor in hepatocellular carcinoma. CAF targeting was achieved by functionalization of the liposome with FH peptide (FH-SSLNav), which displayed an extremely high affinity to tenascin-C [61]. Tenascin-C is a tumor-specific extracellular matrix highly expressed in most solid tumors and mainly secreted by CAF. Tumor exhibited a high-level expression of tenascin-C, which was found to colocalize with CAF. Once in CAF, Navitoclax was released by nanoliposomes in slow rate and inhibited the Bcl-2 proteins, thus inducing apoptosis in CAF at very low dosage. FH-SSLNav revealed efficacy in CAF-induced death, while being much less effective in tumor cells or healthy tissues. Moreover, the treatment led to improved nanoparticles penetration in solid tumor due to modulation of the TME [62]. 

### 3.3. Regulation of CAF Function 

The third approach to modulate TME through direct CAF targeting deals with regulation of CAF function. Indeed, beyond complete ablation of CAFs, some preclinical research has attempted the metabolic targeting of tumor stroma, with the aim to reprogram CAF identity and enhance therapeutic performance of currently available chemotherapeutics.

Major studies for regulation of CAF function have been pursued by Huang group in desmoplastic tumors (i.e., bladder and pancreatic carcinomas) and they assumed to be effective also in other cancer subtypes (i.e., breast cancer) as long as they are desmoplastic [63,64,65]. The first strategy exploits the suppression of Wnt16 in CAF. Indeed, Wnt16 is one of the major mitogenic growth factors and its downregulation improves the antitumor effect of cisplatin in resistant cancers. By developing a Lipid-calcium phosphate (LCP) nanoparticle loaded with cisplatin, the authors observed that the off-target exposure of these nanoparticles induced Wnt16 secretion by CAF. This resulted in stroma reconstitution and onset of cisplatin-resistance, which was reverted by the administration of LCP NP loaded with the anti-Wnt16 siRNA [63]. Wnt16 downregulation was also obtained with LCP NP loaded with the prodrug quercetin, demonstrating its capability to downregulate Wnt16 levels, reduce the number of CAF, normalize the collagen content in tumor tissue and improve nanoparticles delivery into the tumor [64]. Another cunning approach exploits off-targeting capability of LCP nanoparticle coated with Protamine (LPD) to mediate the transfection of CAF with a cytotoxic protein. A plasmid DNA encoding the secretable TNF-related apoptosis-induced ligand (sTRAIL) was formulated in LCP NP and intravenously administered in mice bearing a desmoplastic tumor. As a result of off-target distribution, LCP NP accumulated in CAFs and induced production of sTRAIL. The produced protein was secreted and induced apoptosis in surrounding cells. Moreover, the production of sTRAIL induced also a change in CAF activation, reverting them to a quiescent state, which resulted in inhibition of tumor growth [65]. Despite the low specificity of this delivery strategy, no significant side-effects have been reported, since noticeable morphological changes were not detected in organs where LCP-sTRAIL were distributed. Indeed, a reason can be attributed to the low level of sTRAIL plasmid expression in these organs [65].

Interesting crucial pathway to be targeted when one aims at regulating CAF function is represented by Sonic Hedgehog. A strategy to reduce proliferation and number of CAF was studied in a xenograft mouse model of breast cancer, by using a Sonic Hedgehog inhibitor, vismodegib. Vismogedib targeted CAF with the aim to improve drug delivery. Results from this study suggested that CAF depletion was effective in remodelling the TME, thus promoting fluid stress alleviation. Moreover, the combination of vismodegib with two clinically approved nanoparticles, such as Abraxane and Doxil, improved the treatment efficacy and overall survival in selected patients [66].

Superparamagnetic iron oxide nanoparticles (SPION) were tested to modulate differentiation of human pancreatic stellate cells (hPSCs) into CAF-like myofibroblasts. Indeed, Relaxin-2 is a hormone belonging to the insulin superfamily, able to resolve fibrosis by stimulating MMPs production and inhibiting Smad2/3 phosphorilation, through its binding with the Relaxin Family Peptide Receptor type 1. However, this protein displays small size, short circulation half-life and undesired systemic vasodilatation, which limit its direct application. To solve this issue, the endogenous hormone relaxin-2 was conjugated to SPION, displaying inhibition of Smad2 signaling pathway and blocking hPSCs differentiation into CAF. The inhibition of hPSCs differentiation reduced cytoskeleton marker expression, ECM production and TGF-β-induced contractility of hPCSs. In addition, when combined with gemcitabine, the treatment enhanced the therapeutic efficacy of chemotherapy, resulting in a significant blockade of tumor growth [67].

## 4. Nano-Strategies to Modulate CAF-Instructed Tumor Microenvironment

### 4.1. Remodeling the Extracellular Environment

The ability to control local remodelling of ECM is a critical function of CAF and a feature of paramount importance during the desmoplastic reaction occurring in many breast carcinomas. CAF synthesize and secrete ECM components, cytokines and growth factors that create a favorable support for tumor progression and a physical barrier to several drugs [28]. Dynamic stromal alterations may induce tissue stiffening and increased tension, which have been associated with poor outcome in patients with solid tumors, including breast cancer [26]. Moreover, collagen-rich stroma may induce EMT and promote migration and invasion of breast tumor cells, thus supporting a crucial relevance for tumor environment when approaching novel therapeutic strategies [68]. To face this issue, some preclinical research has counteracted the excessive production of stromal collagen and hyaluronan either through regulation of their secretion by CAF or enzymatically disrupting ECM. 

Losartan, an angiotensin inhibitor, was assembled with C_16_-N peptide hydrogels and injected in a murine model of triple negative breast cancer, where it inhibited collagen I synthesis by CAF [46]. While acting as a local and sustainable depot of drug, the formulation significantly improved the intratumoral accumulation and penetration of PEGylated doxorubicin-loaded liposomes, which were administered as combine therapy. Through physical action on CAF-controlled ECM, C_16_-N/losartan improved drug delivery and vascular perfusion in breast tumor, thereby reducing growth of primary tumor and lung metastasis, as compared to single chemotherapy. In another study, recombinant human hyaluronidase, an enzymatic agent degrading hyaluronic acid, was conjugated to the surface of PLGA NP and pegylated to avoid rapid clearance [47]. Due to ECM degradation, rHuPH20-conjugation improved the intratumoral accumulation of the nanoparticles in 4T1 syngeneic mouse breast tumors by fourfold, as compared to bare nanoparticles. When loaded with doxorubicin, the hyaluronidase-nanoconjugate was able to reduce breast tumor growth in vitro and in vivo, by showing improved efficacy at low dosage as compared to unconjugated nanodrug [47]. ECM-reducing treatments may also increase blood vessels density and revert tumor hypoxia, thus normalizing tumor vasculature and enhancing nanodrug perfusion through EPR [46]. With this perspective, Gong et al. showed enhanced tumor permeation of nanomicelles for PDT when coupled with hyaluronidase for treatment of 4T1-bearing Balb/c mice. The anti-ECM/PDT combined therapy induced increased tumor uptake of the nanomedicine and amplified antitumor efficacy in both breast primary tumor and metastatic lymph nodes [69]. Promising targets for exploitation in nano-mediated tackling of the TME also include tenascin-C, an ECM glycoprotein mainly produced by CAF and highly expressed in breast tumors. The tenascin-C ligand sulfatide was used as targeting moiety for lipid perfluorooctylbromide nanoparticles in order to produce a breast cancer delivery nanovehicle for paclitaxel. Nanoparticles administration in EMT6 mouse model achieved increased accumulation of paclitaxel in breast cancer tissue and remarkable tumor inhibition as compared to free drug or untargeted nanoparticles [48]. 

If ECM disruption improves anticancer drug penetration, reduces hypoxia and overcomes chemoresistance, on the other hand reduced tumor ECM may weaken the physical barrier that confine the tumor at its site of onset, thus allowing formation of neovessels and favoring cancer cells migration and metastasis [70]. For this reason, some research has been devoted to production of artificial ECM. Inspired by the self-assembled formation of natural ECM, Hu et al. developed nanofibers-transforming nanoparticles that mimicked laminin, a component of ECM [49]. Building block for the artificial ECM was laminin-mimic peptide 1 made from (i) hydrophobic bis-pyrene unit for nanoparticle formation, (ii) peptide scaffold for fibers structure, (iii) specific targeting peptide sequence (i.e., RGD/YIGSR) for binding to cancer cells, as well as natural laminin does. Intravenously injected nanoparticles (1-NP) in MDA-MB-231-bearing mice accumulated at the tumor site through combined EPR effect and active targeting. Upon binding to the receptor, 1-NP transformed into nanofibers of artificial ECM that surrounded the tumor mass for over 72 hours post-injection. 1-NP efficiently restrained migration of MDA-MB-231 cells in vitro and induced remarkable inhibition of lung metastatic rate in murine model of highly metastatic breast cancer [49]. In order to block the metastatic spread of cancer cells, mimics of metalloproteinase (MMP) substrates have also been used to counteract the activity of MMPs highly expressed in a variety of tumors, including breast cancer [71]. An example is represented by marimastat (MATT), a broad-spectrum synthetic enzyme inhibitor that achieves great inhibition of collagenases, gelatinases and MMPs even at a nanomolar concentration [72]. (MATT)-loaded thermosensitive liposomes (LTSLs) were assembled with hyaluronic acid-paclitaxel (HA-PTX) prodrug to achieve dual targeting of extracellular TME and cancer cells [50]. Hybrid nanoparticles (HNPs) released their payload upon mild hyperthermia in the TME, where HA-PTX could enter and kill cancer cells, while MATT inhibited MMP activity, reduced activation of CAF and slowed down cancer cells migration. As a result, treatment of 4T1-tumor-bearing mice with HNPs reduced tumor volume, inhibited angiogenesis and decreased lung metastases.

### 4.2. Orchestrate an Anti-Breast Cancer Immune Response

CAF contribute to shape the immune cells in tumors by secreting proinflammatory cytokines and chemokines, notably TGFβ, IL-6 and CCL2, to recruit immunosuppressive cells into the tumor stroma and reject effector T cells [73,74]. The immunosuppressive TME drastically limits the promises of effective immunotherapeutics and checkpoint inhibitors, which have risen new hope for the treatment of several malignant tumors [75]. Overcoming immune suppression in the tumor is of fundamental importance for effective cancer treatment. As already mentioned, therapeutic nano-strategies aimed at targeting and ablating stromal CAF provide great benefit for tumor eradication, by both enabling access of cytotoxic T-cells and by reducing immunosuppressive signals in the TME (see Section 3). However, CAF eradication may not be enough, as pro-tumorigenic cytokines and chemokines are produced by other cell types beyond CAF, here including tumor cells, pericytes, endothelial cells, and adipocytes, which could somehow compensate for CAF absence [76]. The common features of some components of the TME may suggest that targeting mediators of the intercellular communication among CAF, tumor cells, vascular endothelial cells, neutrophils, dendritic cells, T-cells, and macrophages would lead to successful anticancer applications and complement other treatment options. To this purpose, some preclinical studies have proposed to directly modulate the immunosuppressive factors in the TME by nano-mediated delivery of immunotherapy. 

As an example, Feng et al. have generated a dual-activatable binary cooperative prodrug nanoparticle, termed BCPN, which contains self-assembled PEG-grafted oxaliplatin prodrug and a disulphide bond-cross-linked homodimer of NLG919 [51]. Such a nanoparticle was proposed as a platform for codelivery of anticancer drug (OXA) and potent inhibitor of IDO-1 (NLG919), an enzyme implicated in tumor immunosuppression through its ability to limit T-cell function and engage mechanisms of immune tolerance [77]. Due to high sensitivity to the acidic pH of the TME, BCPN showed a negative to positive surface charge reversion for improved tumor penetration. The OXA prodrug and NLG919 dimer were both released following the intracellular reductive microenvironment. OXA induced adaptive antitumor immunogenicity by triggering immunogenic cell death of the tumor cell, while NLG919 inactivated IDO-1, thereby inhibiting intratumoral infiltration of T-regs. Once administered to 4T1-breast-tumor-bearing mice, BCPN promoted sustained and enhanced antitumor immune response as compared to combine treatment with free OXA and NLG919, thus resulting in long-term tumor regression [51]. The same strategy was adopted by Lu et al., who designed a dual-delivery liposome for anticancer drug and IDO-1 inhibitor [52]. They constructed a phospholipid-conjugated indoximod (IDO-1 inhibitor, IND) prodrug that self-assembled into a lipid bilayer liposome, and performed doxorubicin loading as a second step. NPs injected in an orthotopic 4T1 tumor model dramatically improved the pharmacokinetics and tumor concentration of the two drugs induced immunogenic cell death as a result of DOX activity on cancer cells and recruitment of CD8+ cytotoxic T lymphocytes, with disappearance of T-regs, thus showing effective immune response. The authors further combined the DOX/IND-liposome treatment with an anti-PD-1 antibody and showed synergistic efficacy with immune checkpoint blocking agents. Additional example of combined nano-immunotherapy and -chemotherapy is represented by dual pH-responsive multifunctional nanoparticle for co-loading of the Toll-like receptor agonist resiquimod (R848) and the chemotherapeutic drug doxorubicin (HA-DOX/PHIS/R848) [53]. Tumor active targeting was achieved by doxorubicin conjugation to hyaluronic acid, a ligand for breast cancer cells expressing CD44. R848 is a Toll-like receptor agonist that promote maturation of DCs. The pH-dependent hydrophobic/hydrophilic transformation of NP triggered the disintegration of the core, with release of R848 to exert stimulation and maturation of dendritic cells, while DOX was specifically internalized by cancer cells by receptor-mediated endocytosis. Immunoregulation of DCs activity of NP was assessed in vitro as production of type I interferon and proinflammatory cytokines. Then, intravenous injection of HA-DOX/PHIS/R848 NPs in a murine model revealed significant inhibition of tumor growth compared to both free DOX and free R848, thus demonstrating the synergistic effects of the combined nano-drugs on breast cancer [53].

An interesting study on lung carcinoma documents the exploitation and modulation of neutrophils infiltration to mediate the transport of NPs across the tumor vessel barrier [78]. Gold nanorods were linked to anti-CD11b antibodies (GNRs-CD11b), which target activated neutrophils in the peripheral blood. After photosensitization, acute inflammation can be induced at the tumor site, thus promoting tumor infiltration by neutrophils. The neutrophils, loaded with NPs, infiltrated the tumor, significantly increased NP accumulation and enhanced efficacy of photothermal therapy.

Another strategy could be the adoption of nanoparticles as vehicle for tumor-associated antigens to stimulate antitumor T-cell-mediated immune response. Indeed, PLGA NP were loaded with tumor lysate obtained from fresh breast tumor resections and used to stimulate dendritic cells isolated from human peripheral blood mononuclear cells [54]. Tumor lysate-loaded NPs triggered a more efficient maturation of monocyte-derived DC compared to either tumor lysate or NPs alone, as assessed by immunophenotyping and cytokine release. They also showed a capacity to stimulate naive autologous T helper cells through matured DC. 

Quite recently, Shen et al. produced a nano-delivery system for a gene encoding an antibody-like protein to trap IL-10 in the TME [55]. Indeed, high expression of IL-10 was associated with poor survival in pancreatic and triple-negative breast cancer patients [79]. Therefore, transient expression of IL-10 trap through NP intratumoral delivery could change cytokines and tumor-infiltrating lymphocytes within the TME, resulting in significant antitumor efficacy. The plasmid encoding IL-10 trap was encapsulated into liposome-protamine-DNA (LPD) and administered to 4T1-bearing mice. IL-10 trap alone significantly reduced tumor growth and enhanced median survival over one month, indicating efficacy in immunosuppressive triple negative breast cancer [55].

## 5. Prospective Advancement for Clinical Translation

TME plays a key role in most of tumour progression processes, including proliferation, invasion, and neoangiogenesis [3]. Stroma is not significant only from a biological point of view, but it has also strong clinical implications. Indeed, CAF can affect chemosensitivity, protecting cancer cells from cytotoxic drugs with obvious limitations in anticancer efficacy, particularly in breast cancer subtypes in which an intense cross-talk between tumor cells and microenvironment is present, such as basal-like malignancies [80]. An unsolved issue in clinical management of breast cancer is the possibility to enhance antitumor immunity against cancer cells, and immune-modulating treatments has recently gained a strong relevance both in adjuvant and in neoadjuvant settings [81,82]. However, CAF are also capable to protect malignant cells from T-cells antitumor response, thus probably limiting the great potential of such immune-therapies, as well as antitumor vaccination strategies [33,83,84]. If we consider such relevance of CAF and the great promise of CAF-targeted strategies, it is surprising that stroma-targeted therapy has been poorly explored in clinical trials. Two main reasons explain this lacking. First, it is not clear how to specifically target CAF. FAP has been considered a promising target for CAF, being expressed in over 90% of these stromal cells [85]. However, on the other hand, FAP is also over-expressed in multipotent bone marrow stem cells, thus explaining the overwhelming myelotoxicity observed when this strategy was assessed in pilot clinical studies [86,87]. Preliminary clinical trials have substantially demonstrated no efficacy of anti-FAP strategy, both by direct targeting using the monoclonal antibody sibrotuzumab in lung and colorectal cancer [88,89], and by inhibiting the enzymatic function of FAP using talabostat [90,91]. A fortunate pre-clinical exception is the use of a DNA-based FAP vaccine, which showed an excellent anti-stromal CD8-mediated immunity, with subsequent restoring of chemosensitivity in a mouse model of breast cancer [92]. However, significant myelotoxicity due to over-expression of FAP in the bone marrow has currently limited the clinical exploitation of FAP for CAF targeting. Two surface markers specifically linked to pro-tumorigenic CAF have been identified: CD10 and GPR77 [93,94]. These targets promise a higher specificity for CAF, and a monoclonal antibody toward GPR77 has demonstrated enhanced chemosensitivity and reduction of tumor stem cells in a patient-derived model of breast cancer [30]. Secondly, it is not clear which CAF have to be depleted. A tout-court depletion of CAF, indeed, might paradoxically promote cancer progression, since CAF within certain subsets or within certain stages of tumorigenesis express an anti-cancer profile [95]. Recently, it has been suggested that quiescent fibroblasts, upon development of cancer, might differentiate into cancer-restraining (F1 subtype) and cancer-promoting (F2 subtype) CAF [22]. Furthermore, “secretory CAF” have been identified as a third possible differentiation, leading itself to cancer survival vs. cancer apoptosis pathways, based on the secretome context. For example, some F3 CAF produce ECM-degrading proteases which facilitate motility and invasion of cancer cells [96]. However, on the other hand, it has been observed that CAF, upon secretion of TGFβ, inhibit cancer development in early stages but the same pathway may stimulate cancer in advanced stages [97]. Furthermore, tumor-restraining CAF may increase antitumor immunity, by secretion of immunomodulatory cytokines such as IL-10, TNF, and IL-6, aiding in recruitment of macrophages and T lymphocytes, thus converting an immune-suppressive into immune-stimulating cancer microenvironment [98]. Recently, novel approaches including single-cell RNA sequencing have revealed that distinct subtypes of CAF co-exist within TME, with different phenotypes and functions [99]. Thus, the concept that cancer-related stroma is not heterogeneous like cancer cells has been profoundly revised, and precision medicine is required also in CAF-targeted anticancer strategies.

Turning CAF “from foes to friends” may, therefore, establish as the most appropriate treatment to target the stroma, since possible pitfalls of complete depletion of CAF [8], which may limit clinical translation of this strategy, might be avoided. Under this innovative perspective, nanomedicine represents an added value for CAF-targeted anticancer treatments. Indeed, nanoparticles are optimal drug delivery systems since they present several advantages in cancer therapy. First, they allow their cargo to follow the so-called EPR effect, by which molecules of certain sizes tend to accumulate in tumor tissue much more than they do in normal tissues. This property not only leads to increased drug accumulation in the tumor mass, but also avoids off targeting and associated side effects, which could rise hope on reduced myelotoxicity when targeting FAP by appropriate nano-vehicles. Second, nanoparticles can be loaded with specific drugs focused on particular cell pathways and possibly acting on different subcellular districts. Precision medicine should rely not only on accurate targets, such as in the case of targeted monoclonal antibodies which can exert their anti-stromal activity by immune-mediated CAF depletion. Additionally, accurate effects are needed, for example to convert a pro-tumorigenic into an anti-cancer CAF profile by acting on CAF-mediated signaling, such as the JAK1-STAT3 pathway [100], or by genetically modifying CAF themselves to make them quiescent or even to allow in situ production of pro-apoptotic mediators, as recently proposed [65]. An accurate targeting might be ensured by novel nano-drugs, but the question is which target should be used for properly addressing such strategies in activated CAF and avoiding cytotoxic effects on normal/quiescent or F1 fibroblasts. As previously stated, FAP showed encouraging results, but its over-expression in bone marrow may also represent a relevant drawback. CD10 and GPR77 could be more specific and accurate as hallmarks of activated cancer-promoting CAF, and further research should address their usefulness as targets for nano-delivery of cytotoxic drugs or, possibly, of CAF-reprogramming strategies. Another aspect to be considered is the availability of concurrent drugs delivery and multiple contemporary anticancer effects thanks to nano-therapy, making possible to act both on cancer cells and on TME at the same time, thus maximizing the anticancer effects and reducing chemoresistance. Notably, a double-threaded link is present between nanomedicine and TME. On one hand, nano-delivery may overcome current clinical limitations and acts on stromal cells to enhance chemosensitivity and immune response. However, on the other hand, TME strongly affects the capability of nanoparticles to reach the tumor sites, thus impacting on anticancer efficacy of delivered drugs, particularly in the complex stroma of breast cancer [101]. A combinatorial therapy may therefore act on CAF and other stromal compounds, allowing cytotoxic drugs specifically delivered by nanoparticles to enhance their effect.

Several potentialities are promised by nanomedicine for specific and multi-acting cancer treatments, but the pivotal issue is whether nanomedicine is clinically transferable in the next future. A first great limitation is the lack of suitable preclinical models which accurately resemble spatially and biologically human breast cancer and its complex interaction with surrounding microenvironment. Clinical translation cannot be independent from this point, as widely demonstrated in current research, since the great majority of promising drugs and therapeutic strategies developed in preclinical studies do not reach clinical relevance [102]. Furthermore, preclinical reliable models of TME is even more difficult, since spatial issues might be fundamental to properly resemble the real biological situation [103]. Other major questions arise about safety, production standardization, and costs as well as potential toxicities before the use of targeted nanoparticles in clinical practice, and research in this field needs to be further accelerated.

## Figures and Tables

**Table 1:** Nano-strategies to target CAF and remodel the TME in preclinical models of breast cancer.

Nanoparticle	Payload	Effect on	Breast Cancer Model	Antitumor Effect	Ref.
Z@FRT-scFv	ZnF_16_Pc	FAP + CAF	Orthotopic 4T1 tumor	CAF eradication by PDT	[42,43]
CAP-NP	PTX (or other hydrophobic drugs)	FAP + CAF and surrounding cells	MCF-7 xenograft	Enhanced local drug accumulation	[44]
C_16_-N/losartan hydrogel	Losartan	Angiotensin	E0771 and 4T1 mouse models	CAF inhibition + ECM remodeling	[45,46]
DOX-HPEG-PH20-NP	rHuPH20 + DOX	Hyaluronic acid + cancer cells	4T1 syngeneic breast tumor	ECM remodeling + chemotherapy	[47]
PTX-SNPs	Sulfatide + PTX	Tenascin-C + cancer cells	Murine breast cancer EMT6	Enhanced chemotherapy	[48]
1-NP	Laminin-mimic peptide 1	TME	MDA-MB-231 tumor model	Reduced metastases by artificial ECM formation	[49]
HNP liposomes	Marimastat + HA-PTX prodrug	MMP + CD44+ cancer cells	orthotopic 4T1 tumor	ECM remodeling + chemotherapy	[50]
BCPN	OXA prodrug + NLG919	IDO-1 + cancer cells	orthotopic 4T1 tumor	Immunotherapy + chemotherapy	[51]
DOX/IND-liposome	Indoximod + DOX	IDO-1 + cancer cells	orthotopic 4T1 tumor model	Immunotherapy + chemotherapy	[52]
HA-DOX/PHIS/R848	Resiquimod + DOX	DC + CD44+ cancer cells	4T1 tumor-bearing mice	Immunotherapy + chemotherapy	[53]
PLGA NP	Tumor antigens	DC	Tumor and blood samples from breast cancer patients	Immune-stimulation	[54]
LPD	Plasmid encoding IL-10 trap	TME	Orthotopic 4T1 triple-negative model	Immunotherapy	[55]

## Abbreviations

α-SMA	Alpha-smooth muscle actin
Bcl-2	B-cell lymphoma 2
BCPN	Binary cooperative prodrug nanoparticle
CAF	Cancer-associated fibroblasts
CAP	Cleavable amphiphilic peptide
CCL2	Chemokine C-C motif ligand 2
CD	Cluster of differentiation
CPP	Cell-penetrating peptide
CXCL	C-X-C motif ligand
DC	Dendritic cells
DOX	Doxorubicin
ECM	Extracellular matrix
EMT	Epithelial to mesenchymal transition
EPR	Enhanced permeability and retention
FAP	Fibroblast activation protein
GNRs	Gold nanorods
HA	Hyaluronic acid
HNP	Hybrid nanoparticle
hPSCs	Human pancreatic stellate cells
IDO-1	Indoleamine 2,3-Dioxygenase 1
IL	Interleukin
LCP	Lipid-calcium phosphate
LPD	Liposome-protamine-DNA
LTSLs	Loaded thermosensitive liposomes
MATT	Marimastat
MMP	Metalloproteinases
NP	Nanoparticles
OXA	Oxaliplatin
PEG	Polyethylene glycol
PDGF	Platelet-derived growth factor
PDT	Photodynamic therapy
PLGA	Poly(lactic-co-glycolic) acid
PNP	Peptide nanoparticles
PS	Photosensitizer
PTX	Paclitaxel
QDs	Quantum dots
ROS	Reactive oxygen species
SPION	Superparamagnetic iron oxide nanoparticles
siRNA	Short interfering RNA
TGFβ	Transforming growth factor beta
TME	Tumor microenvironment
TNF	Tumor necrosis factor
TRAIL	TNF-related apoptosis-induced ligand
VEGF	Vascular endothelial growth factor
